# Exploring Mechanisms of Biofilm Removal

**DOI:** 10.4172/2161-1122.1000371

**Published:** 2016-04-25

**Authors:** Karan Sahni, Fatemeh Khashai, Ali Forghany, Tatiana Krasieva, Petra Wilder-Smith

**Affiliations:** Beckman Laser Institute, University of California, Irvine, California 92612, USA

**Keywords:** Biofilm, Dental plaque, EPIEN dental debriding solution, Oral hygiene

## Abstract

**Objective:**

The goal of this study was to evaluate the effects of a novel anti-plaque formulation on oral biofilm removal. Specific aim was to elucidate the role of 2 potentially complementary mechanisms on dental biofilm removal using EPIEN Dental Debriding Solution (EDDS) like desiccating action leading to denaturation and destabilization of plaque and mechanical removal of destabilized plaque through forceful rinsing action

**Materials and Methods:**

25 extracted teeth, after routine debriding and cleaning, underwent standard biofilm incubation model over 4 days. Then samples were randomly divided into 5 groups of 5 teeth each, treated and stained with GUM^®^Red-Cote^®^ plaque disclosing solution and imaged. Samples were subsequently treated with HYBENX^®^ Oral Decontaminant. Group 1 samples were treated with a standardized “static” water dip exposure following biofilm incubation. Samples in Group 2 were given a standardized “dynamic” exposure to a dental high pressure air/water syringe for 20 s. Group 3 samples were exposed to a standardized “static” application of test agent (30 s dip rinse) followed by a standardized “static” water rinse (30 s dip rinse). Samples in Group 4 were given both the standardized “static” application of test formulation followed by the standardized “dynamic” exposure to a dental high pressure air/water syringe. Finally, samples in Group 5 were treated with a standardized “dynamic” application of test agent (20 s high pressure syringe at 10 ml/s) followed by the standardized “dynamic” exposure to a dental high pressure air/water syringe.

**Results:**

The MPM images demonstrated that the water dip treatment resulted in the persistence of an almost continuous thick layer of biofilm coverage on the tooth surface. Similarly, test agent dip treatment followed by water dip only removed a few patches of biofilm, with the majority of the tooth surface remaining covered by an otherwise continuous layer of biofilm. Samples exposed to air/water spray alone showed some disruption of the biofilm, leaving residual patches of biofilm that varied considerably in size. Test agent dip treatment followed by air/water spray broke up the continuous layer of biofilm leaving only very small, thin scattered islands of biofilm. Finally, the dynamic test agent spray followed by air/water spray removed the biofilm almost entirely, with evidence of only very few small, thin residual biofilm islands.

**Conclusion:**

These studies demonstrate that test agent desiccant effect alone causes some disruption of dental biofilm. Additional dynamic rinsing is needed to achieve complete removal of dental biofilm.

## Introduction

Biofilm consists of a community of microorganisms embedded in a self-produced extracellular polysaccharide matrix that develop on substratum or submerged surfaces such as living tissues, tooth surfaces and medical devices [1]. The oral cavity harbors one of the most diverse microbiomes in the human body, providing several distinct and hospitable microbial habitats such as the teeth and gingival sulcus [2]. The resident oral microflora consists of a dynamic multi-species community of bacteria that grow and adhere to the enamel pellicle and mucosal surfaces [3]. If left *in situ*, oral biofilm, in the form of supragingival and subgingival plaque, may have a wide range of effects including the formation of dental calculus, dental demineralization and caries, gingival inflammation, and periodontal disease [4]. Studies in the USA and the UK suggest that some degree of gingivitis affects 50–90% of the adult population [5]. Furthermore, a recent publication from the National Health and Nutrition Examination Survey (NHANES) found that 47% of adults in the US suffer from periodontitis [6]. Oral biofilm can affect systemic health as well, providing added impetus for more effective approaches to oral hygiene.

Currently, oral biofilm control is primarily accomplished through the use of dentifrice containing compounds such as detergents, abrasives and antimicrobials, which achieve their effects in conjunction with mechanical tooth brushing [7]. If biofilm accumulation and growth can be reduced and its re-aggregation discouraged, this will result in improved gingival health [8]. Conversely, ineffective plaque control is directly implicated in gingival inflammation and eventually in destructive chronic periodontitis [9]. Despite its essential role in the prevention of gingivitis and periodontitis, and often considerable efforts at oral hygiene by patients, effective and stable plaque control remains elusive to many individuals [10–12]. Accordingly, a multitude of novel anti-plaque formulations are under investigation for their ability to remove oral biofilm and to prevent its re-accumulation [11,13,14].

The goal of this *ex vivo* study was to test the efficacy of a novel dental debriding formulation, HYBENX^®^ Oral Decontaminant (EPIEN Medical Inc., St. Paul, MN, USA), for biofilm removal and control. Its primary mode of action is hypothesized to be through chemical desiccation or dehydration, leaving a denatured and destabilized biofilm which can then be removed by irrigation. In this pilot study, 3D Nonlinear Optical Microscopy imaging (NLOM) was used to generate non-contact high-resolution images of the biofilm and tooth surface [15–17].

## Materials and Methods

25 extracted teeth, after routine debriding and cleaning, underwent standard biofilm incubation model over 4 days. Briefly, an artificial mouth model (AMM) was used to mimic the *in vivo* situation as closely as possible. The model consisted of an *in vitro* dental biofilm system with a continuous, open-surface fluid flow [18]. It used a microcosm saliva inoculate [19] and provided a standardized flow of nutrients over the biofilm [20]. Then samples were randomly divided into 5 groups of 5 teeth each, and treated with HYBENX^®^ Oral Decontaminant as indicated below. Samples were stained with GUM^®^Red-Cote^®^ plaque disclosing solution (Sunstar Americas, Inc., Chicago, IL) and then imaged.

- Group 1: Water dip rinse only treatment (30 s) (to map biofilm presence on each sample after 4 day standard incubation model, with dip treatment modeling saliva presence in the mouth).
- Group 2: A standardized “dynamic” exposure to a dental high pressure air/water syringe (20 s) (to identify the role of clinical usage-equivalent mechanical force on biofilm presence).
- Group 3: A standardized “static” application of EDDS (30 s dip rinse) followed by a standardized “static” water rinse (30s dip rinse) (to identify the desiccation effect of EDDS on biofilm presence absent added mechanical rinsing effects).
- Group 4: A standardized “static” application of EDDS (30 s dip rinse) followed by a standardized “dynamic” exposure to a dental high pressure air/water syringe (20 s) (to identify the role of clinical usage-equivalent mechanical force on biofilm after EDDS desiccation treatment).
- Group 5: A standardized “dynamic” application of EDDS (20 seconds from a high pressure syringe @ 10 ml/s) followed by a standardized “dynamic” exposure to a dental high pressure air/water syringe (20 s) (to identify the role of clinical usage-equivalent mechanical force during and after EDDS desiccation treatment on biofilm).

### Imaging

Non-Linear Optical Microscopy (NLOM) imaging techniques were used to image treatment effects on biofilm. NLOM uses exogenous and endogenous fluorescence properties that allow for intact biological samples to be visualized at specific surface and subsurface locations at high-resolution [15,16]. NLOM generated images consisting of 3D views generated from stacks of 2D scans. These 3D images can be manipulated and sectioned optically in any plane, allowing for accurate visualization, mapping and measurement of structures such as biofilm or the tooth surface [15]. In this study, standardized evaluation criteria included: (1) biofilm thickness measurements at 3 standardized locations per sample, (2) biofilm content and cohesion as a descriptive measure and (3) measurement of the percentage of each tooth surface that was covered by biofilm.

## Results

### Sample appearance

Extracted tooth samples from Group 1 (water dip treatment) and Group 3 (test agent dip/water dip treatment) appeared to be covered by a dull layer of plaque, visible to the naked eye on all samples. Samples in Group 2 (air/water spray treatment) appeared much less dull, and had a glossy sheen on the enamel surface. Finally, extracted tooth samples in Group 4 (test agent dip and air/water spray) and Group 5 (dynamic test agent spray and air/water spray) appeared shiny, white and clean to the naked eye.

### Imaging data

**Figure 1:**
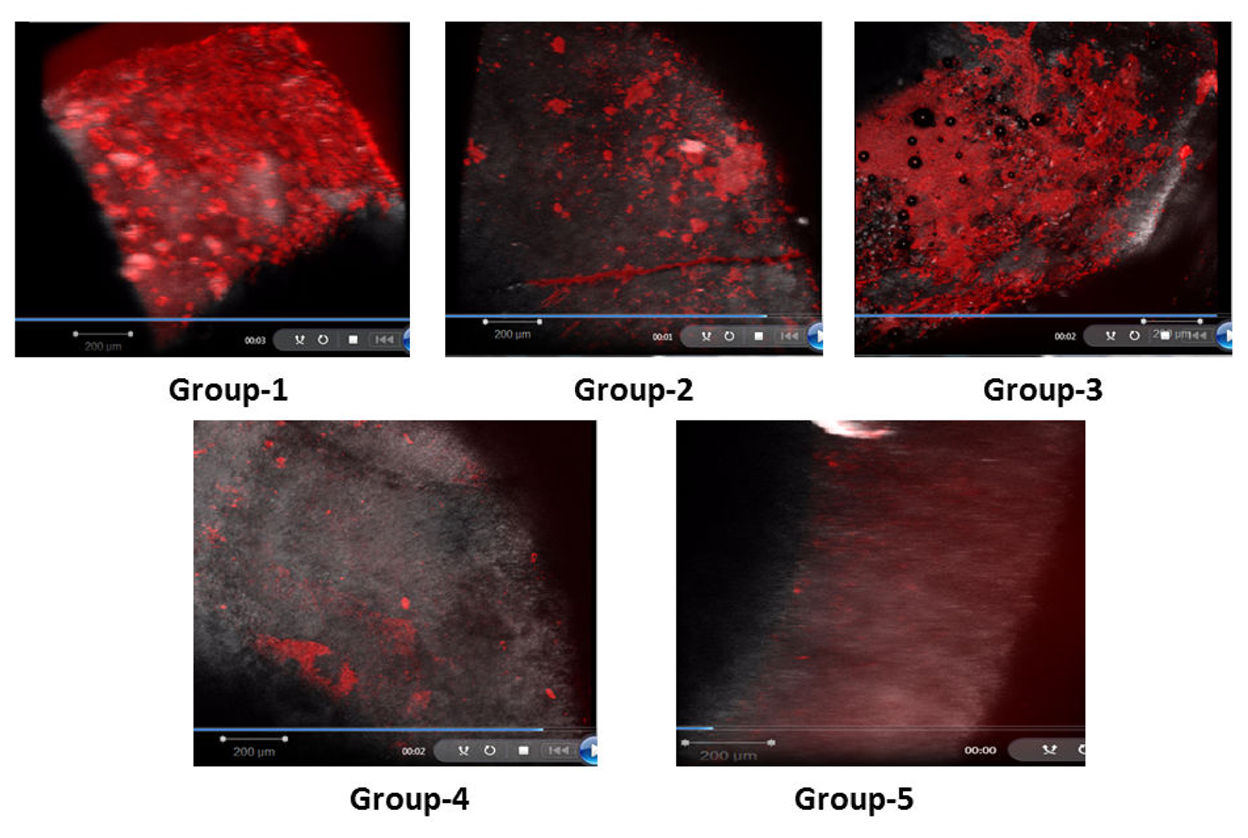
Non-linear optical microscopy (NLOM) images of tested samples. A: Group 1 - Water dip; B: Group 2 - Air/water spray; C: Group 3 - Test agent dip and water dip; D: Group 4 - Test agent dip and air/water spray; E: Group 5 - Dynamic test agent spray and air/water spray.

Using NLOM, sample enamel surface and biofilm coverage were clearly visible. In the NLOM images, the biofilm appeared as a bright red fluorescence signal, while the enamel surface was visible as a gray/purple fluorescing structure (Figure 1).

NLOM images from samples within the same group were consistent and very similar, whereas the appearance of the biofilm and tooth surface varied considerably between groups (Figures 1A–1E). Figure 1A depicts a Group 1 tooth sample following water dip treatment. A bright red, thick, nearly uniform red fluorescent biofilm layer is visible on the enamel surface. The presence of this continuous thick layer of biofilm shows the relative ineffectiveness of the water dip treatment alone in plaque removal. Figure 1B shows a representative image for Group 2 samples (dynamic exposure to a dental high pressure air/water syringe for 20 s). Predominantly a gray fluorescence signal from enamel is visible, with frequent islands of red biofilm fluorescence, indicating the presence of residual areas of plaque on the enamel surface following air/water spray treatment. Clearly the air/water spray treatment caused some disruption of the biofilm. In Figure 1C, an image of a tooth sample following both test agent and water dip treatment is presented, showing a nearly continuous layer of red fluorescent biofilm coverage except for a few patches of biofilm-free gray enamel. A representative image of a Group 4 sample (EDDS dip rinse followed by dental high pressure air/water syringe) is shown in Figure 1D. Extensive biofilm removal is evident, with some minor, scattered residual islands of biofilm. Finally, Figure 1E depicts a representative NLOM image of a sample from Group 5 following dynamic test agent spray and air/water spray. The image shows that this treatment combination achieved almost complete biofilm removal.

### Semi-quantification of sample coverage by biofilm

Results shown in Table 1 demonstrate that the dynamic test agent spray in combination with the air/water spray treatment regimen (Group 5) was the most effective in biofilm removal, reducing the total tooth surface coverage by biofilm to approximately 2%. Group 4 treatment protocol resulted in tooth samples with roughly 9% residual biofilm coverage on the enamel surface, while the air/water spray regimen in Group 3 left approximately one-third of the enamel surface covered with biofilm. Finally, samples from Group 3 and Group 1 resulted in the most residual biofilm on the enamel surface at 83% and 92% biofilm coverage, respectively.

## Discussion

In this study, NLOM imaging provided a quick, effective means of mapping biofilm on the tooth surface. NLOM is based on two-photon excited fluorescence (TPEF) which has several advantages over standard fluorescence microscopy, such as the elimination of photo bleaching of fluorescent molecules outside the focus [15,16]. Another important advantage of TPEF over single-photon absorption is that near infrared light is used instead of visible light. Longer wavelengths of light experience less scattering and absorption in biological tissue, allowing for deeper penetration and imaging depth [15,16].

The structure and viability of the biofilm produced by the standard model used in this study model are considered to be comparable to *in vivo* generated biofilm as judged in previous studies by multiple methods including CLSM [21,22]. Indeed, the NLOM images of biofilm generated in this study resembled the appearance of biofilm grown in vivo and imaged *in situ* in other studies. Previously, bacterial biofilm growth has been imaged on bovine enamel slabs using fluorescence *in situ* hybridization (FISH) in conjunction with Confocal Laser Scanning Microscopy (CLSM). This combination of imaging techniques can enable efficient visualization and quantification of biofilm growth on the enamel surface [23]. However, CLSM has some limitations when compared to NLOM, including a very small field of view and heating of the target tissue area [23]. Due to its nonlinear nature, NLOM fluorescence generation is essentially confined to the focal volume, giving high resolution without the need for spatial filtering as in confocal microscopy [23].

NLOM images showed that EDDS alone causes some disruption of dental biofilm. However, additional forceful rinsing is to achieve comprehensive removal of dental biofilm. Samples in Group 4 (EDDS dip and air/water spray) and Group 5 (dynamic EDDS spray and air/water spray) evidenced the highest levels of biofilm removal from the enamel surface, at 91% and 98% respectively. These results confirm the hypothesis that the treatment formulation acts through two mechanisms of biofilm control, which must work in conjunction to adequately reduce plaque accumulation. Previous research confirms the findings of this study that a combination of an antiplaque formulation and mechanical removal is optimal for comprehensive oral biofilm removal [24]. Thus it is common clincial practice to supplement mechanical cleaning with a combination of chemical agents, sonication and irrigation to combat dental plaque [25,26]. It has been shown that plaque removal using sonic power toothbrushes versus traditional manual toothbrushes is superior in reducing overall plaque coverage [27]. Moreover, studies evaluating the efficacy of dental water jets for plaque control confirm that pressurized irrigation removes biofilm [28]. Finally, clinical studies investigating the effects of adding daily oral irrigation to power or manual tooth brushing regimens found that added oral irrigation techniques can enhance the effectiveness of plaque control [29]. Additional *in vivo* investigations are planned to identify the *in vivo* effects of this formulation, and to further elucidate the balance between the 2 mechanisms of action demonstrated in this paper.

## Conclusion

Using novel NLOM imaging techniques the effects of a novel antiplaque formulation were mapped and quantified. The treatment product displayed effective levels of biofilm removal, especially when coupled with mechanical rinsing action. Additional studies, especially *in vivo* investigations, are needed to further elucidate the short-, mid- and long-term role of HYBENX^®^ Dental Debriding Solution in plaque and oral biofilm control.

## Figures and Tables

**Table 1:** Percent of tooth surface coverage by biofilm.

	Group 1 (n=5)	Group 2 (n=5)	Group 3 (n=5)	Group 4 (n=5)	Group 5 (n=5)
**Treatment**	Water dip	Air/water spray	Test agent dip and water dip	Test agent dip and air/water spray	Dynamic test agent spray and air/water spray
**% Tooth surface coverage by biofilm (SD)**	92% (5)	35% (4)	83% (7)	9% (2)	2% (1)
